# Myeloid Dendritic Cells Induce HIV-1 Latency in Non-proliferating CD4^+^ T Cells

**DOI:** 10.1371/journal.ppat.1003799

**Published:** 2013-12-05

**Authors:** Vanessa A. Evans, Nitasha Kumar, Ali Filali, Francesco A. Procopio, Oleg Yegorov, Jean-Philippe Goulet, Suha Saleh, Elias K. Haddad, Candida da Fonseca Pereira, Paula C. Ellenberg, Rafick-Pierre Sekaly, Paul U. Cameron, Sharon R. Lewin

**Affiliations:** 1 Department of Infectious Diseases, Monash University, Melbourne, Victoria, Australia; 2 Centre for Biomedical Research, Burnet Institute, Melbourne, Victoria, Australia; 3 VGTI-Florida, Port St. Lucie, Florida, United States of America; 4 Laboratoire d'immunologie, Centre de Recherche du Centre Hospitalier de l'Université de Montréal, Montréal, Quebec, Canada; 5 Monash Micro Imaging, Monash University, Melbourne, Victoria, Australia; 6 Infectious Diseases Unit, Alfred Hospital, Melbourne, Victoria, Australia; University of Pennsylvania School of Medicine, United States of America

## Abstract

Latently infected resting CD4^+^ T cells are a major barrier to HIV cure. Understanding how latency is established, maintained and reversed is critical to identifying novel strategies to eliminate latently infected cells. We demonstrate here that co-culture of resting CD4^+^ T cells and syngeneic myeloid dendritic cells (mDC) can dramatically increase the frequency of HIV DNA integration and latent HIV infection in non-proliferating memory, but not naïve, CD4^+^ T cells. Latency was eliminated when cell-to-cell contact was prevented in the mDC-T cell co-cultures and reduced when clustering was minimised in the mDC-T cell co-cultures. Supernatants from infected mDC-T cell co-cultures did not facilitate the establishment of latency, consistent with cell-cell contact and not a soluble factor being critical for mediating latent infection of resting CD4^+^ T cells. Gene expression in non-proliferating CD4^+^ T cells, enriched for latent infection, showed significant changes in the expression of genes involved in cellular activation and interferon regulated pathways, including the down-regulation of genes controlling both NF-κB and cell cycle. We conclude that mDC play a key role in the establishment of HIV latency in resting memory CD4^+^ T cells, which is predominantly mediated through signalling during DC-T cell contact.

## Author Summary

Current antiretroviral drugs significantly prolong life and reduce morbidity but are unable to cure HIV. While on treatment, the virus is able to hide in resting memory T cells in a silent or “latent” form. These latently infected cells are rare and thus are hard to study using blood from HIV-infected individuals on treatment. Therefore, it is very important to have laboratory models that can closely mimic what is going on in the body. We have developed a novel model of HIV latency in the laboratory. Using this model we have shown that the presence of dendritic cells, an important type of immune cell that can regulate T cell activation, at the time of infection allows for the infection of resting T cells and the establishment of latency. We have demonstrated that this is predominantly mediated by direct cell-to-cell interactions. Further exploration of the mechanisms behind HIV latency could lead to new ways to treat and possibly eradicate HIV.

## Introduction

Antiretroviral therapy (ART) for the treatment of HIV has led to a substantial reduction in morbidity and mortality; however, ART cannot cure HIV and life-long treatment is required. This is directly due to the persistence of long-lived latently infected cellular reservoirs, that include microglia, astrocytes, macrophages and naïve T cells [1]–[4], however, resting memory CD4^+^ T cells [5]–[7], are considered to be the major contributors. Latently infected resting CD4^+^ T cells are found in blood and tissue sites, including lymphoid tissue and the gastrointestinal tract [7]–[10]. The frequency of latently infected cells is up to ten times higher in tissue than in blood in HIV-infected patients or SIV-infected macaques on suppressive ART [8], [10].

It is unclear how latency is established *in vivo*. However, *in vitro*, latency can be established following survival of an activated CD4^+^ T cell that returns to a resting state carrying integrated virus [5], [11]–[13]. Alternatively, latency has also been established following direct infection of resting cells in the presence of chemokines or following spinoculation [14]–[19]. Dendritic cells (DC) are found throughout the body and interact closely with resting CD4^+^ T cells within lymphoid tissues. Therefore, given the high frequency of latently infected cells in lymphoid tissue, we hypothesised that latency in resting CD4^+^ T cells may result from interactions with DC as CD4^+^ T cells recirculate through lymphoid tissue. Using a novel model of resting CD4^+^ T cells co-cultured with primary DC, we demonstrate that myeloid DC (mDC) induce post-integration latency in resting memory CD4^+^ T cells, which required close DC-T cell contact.

## Results

### Myeloid DC promote HIV latency in non-proliferating CD4^+^ T cells

**Figure 1:**
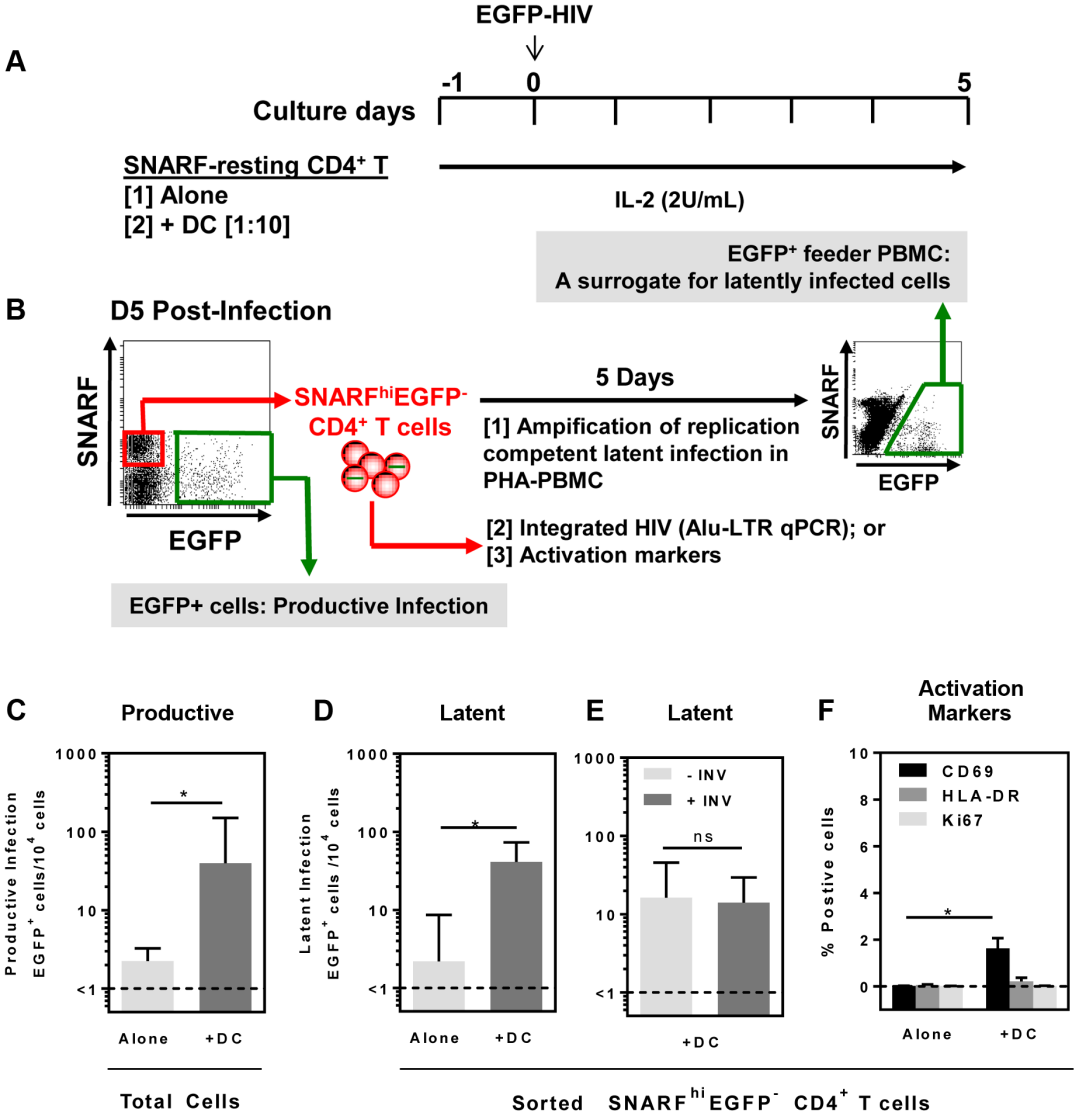
DC-induced latency in resting CD4^+^ T cells. (**A**) Resting CD4^+^ T cells were isolated from the blood of healthy donors and labelled with the proliferation dye SNARF, which decreases in intensity following each round of cell division allowing identification of non-proliferating cells. SNARF-labelled resting CD4^+^ T cells were cultured either alone or with syngeneic blood DC. Following 24 hours of culture, cells were infected with NL(AD8)-nef/EGFP at an MOI of 0.5. All culture media was supplemented with IL-2 (2 U/mL). (**B**) At day 5 post-infection, the number of productively infected (EGFP^+^) cells was determined and the non-proliferating (SNARF^hi^) cells that were not productively infected (EGFP^−^) were sorted. The sorted SNARF^hi^EGFP^−^ cells were stimulated with PHA/IL-2 in the presence of PBMC and cultured for 5 days to amplify any replication competent latent infection. (**C**) Productive infection and (**D**) latent infection following infection of T cells cultured alone (light grey) or in the presence of DC (grey) is shown. (**E**) Latent infection in the presence of DC cultured with (grey) or without (light grey) 0.1 µM Indinavir. (**F**) Expression of the early (CD69; black) and late (HLA-DR; grey) surface activation markers and the intracellular proliferation marker Ki67 (light grey) was quantified by flow cytometry on sorted SNARF^hi^EGFP^+^ CD4^+^ T cells following HIV infection of T cells cultured alone or in the presence of DC. The lower limit of detection of each assay is represented by a dotted line. Columns represent the median of 5 independent experiments and error bars indicate the interquartile range. *P<0.05 (Wilcoxon signed-rank test).

Resting CD4^+^ T cells and syngeneic DC (including the two major blood DC subpopulations, plasmacytoid (pDC) and myeloid (mDC) DC) were sorted from the blood of healthy donors (Fig. S1). Seminaphtharhodafluor-1 (SNARF)-labelled resting CD4^+^ T cells were cultured either alone or co-cultured with DC at a DC: T cell ratio of 1: 10. Following 24 hours of culture, cells were infected with a CCR5-tropic enhanced green fluorescent protein (EGFP)-reporter virus, NL(AD8)-nef/EGFP (multiplicity of infection, MOI 0.5), and cultured for 5 days (Fig. 1 A). Cells were then analysed for expression of EGFP by flow cytometry to quantify productive infection (Fig. 1 B).

In the DC-CD4^+^ T cell co-cultures, we detected a spreading productive infection with the number of infected cells 5 days post-infection significantly greater (median (IQR) = 40 (31, 150) EGFP^+^ cells/10^4^ cells; n = 5) compared to CD4^+^ T cells cultured alone (1.5 (1, 2.5) EGFP^+^ cells/10^4^ cells, p = 0.03; Fig. 1 C). These results were consistent with previous work demonstrating enhanced productive infection of CD4^+^ T cells in the presence of DC [20], [21].

At day 5 post-infection, non-proliferating (SNARF^hi^) CD4^+^ T cells that were not productively infected (EGFP^−^) were sorted (purity was always >99%). Latent virus was quantified in the SNARF^hi^EGFP^−^ CD4^+^ T cells upon stimulation with phytohaemagglutinin (PHA) in the presence of feeder peripheral blood mononuclear cells (PBMC) following a further 5 days of culture (Fig. 1 B). The number of EGFP^+^ cells following stimulation was, therefore, a surrogate measure for the number of latently infected cells in the SNARF^hi^EGFP^−^ CD4^+^ T cells. When SNARF^hi^EGFP^−^ CD4^+^ T cells were sorted from cultures infected in the absence of DC, few latently infected cells were detected (2 (1, 8.5) EGFP^+^ cells/10^4^ cells; n = 5). In contrast, when SNARF^hi^EGFP^−^ CD4^+^ T cells were sorted from the DC-T cell co-cultures a significant increase in the number of latently infected cells was observed (41 (28, 73) EGFP^+^ cells/10^4^ cells; p = 0.03; n = 5; Fig. 1 D). Furthermore, when infections were performed in the presence of the protease inhibitor indinavir, there was no significant difference in the number of latently infected cells, as measured by EGFP expression following co-culture of SNARF^hi^EGFP^−^ with PHA and feeder PBMC (Fig. 1 E). This confirms that a productive, spreading infection was not required to establish latency. Together, these results demonstrate that DC facilitate latent HIV infection in non-proliferating CD4^+^ T cells.

We next asked whether DC-T cell co-culture had activated the SNARF^hi^ CD4^+^ T cells, which allowed for HIV entry. Sorted SNARF^hi^EGFP^−^ CD4^+^ T cells that were co-cultured with DC for 5 days showed signs of early activation with increased expression of CD69 (1.5% (0.1, 2.1); p = 0.02; n = 4; Fig. 1 F). However, these cells did not express either HLA-DR or Ki67 (Fig. 1 F). As expected, resting CD4^+^ T cells that were cultured alone did not express any of the activation markers. These results confirmed that the sorted SNARF^hi^EGFP^−^ cells were non-proliferating, partially activated CD4^+^ T cells.

**Figure 2:**
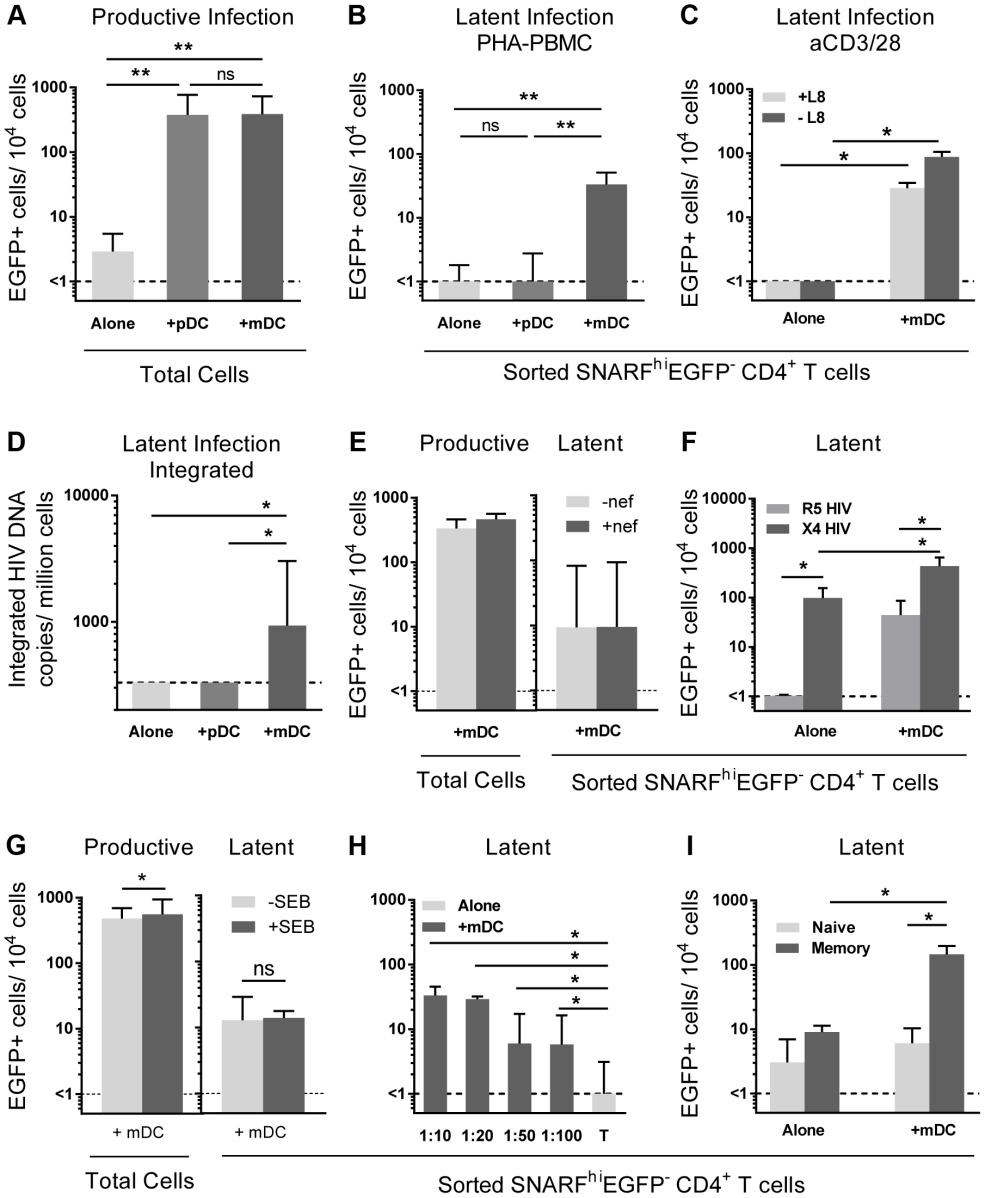
Myeloid DC induce post-integration latency in non-proliferating memory CD4^+^ T cells. SNARF-labelled resting CD4^+^ T cells were cultured alone (light grey) or with syngeneic plasmacytoid (pDC; grey) or myeloid DC (mDC; dark grey). (**A**) Productive infection (EGFP^+^ cells) was determined by flow cytometry on day 5 post-infection. (**B**) Latent infection was quantified in SNARF^hi^EGFP^−^ cells following either addition of PHA-activated PBMC, n = 5; or (**C**) direct activation with anti-CD3/CD28 in the presence or absence of the integrase inhibitor L8. (**D**) Integrated HIV DNA was quantified in the sorted SNARF^hi^EGFP^−^ T cells by Alu-LTR real-time PCR, n = 3. (**E**) Productive and latent infection was determined in SNARF^hi^EGFP^−^ CD4^+^ T cells from mDC-T cell co-cultures following infection with nef-deficient (-nef) or nef-competent EGFP HIV. (**F**) Latent infection was determined in sorted SNARF^hi^EGFP^-^ CD4^+^ T cells, cultured alone or with mDC, following activation with PHA-PBMC. (**G**) Productive and latent infection was determined in SNARF^hi^EGFP^−^ CD4^+^ T cells from mDC-T cell co-cultures with and without Staphylococcus Enterotoxin B (SEB), n = 4. (**H**) SNARF-labelled resting CD4^+^ T cells were cultured alone (light grey) or with syngeneic mDC (grey) at decreasing DC∶T cell ratios and latent infection quantified in sorted SNARF^hi^EGFP^−^ T cells following addition of PHA-activated PBMC, n = 5. (**I**) Resting CD4^+^ T cells were cultured either alone or in the presence of mDC. At day 5 post-infection, SNARF^hi^EGFP^−^ cells were sorted into naïve (light grey) or memory (grey) CD4^+^ T cells and latent infection quantified, n = 5. The lower limit of detection is represented by a dotted line. Columns represent the median of 3–7 donors and error bars indicate the interquartile range. *P<0.05; **P<0.01; ns, not significant (Wilcoxon signed-rank test).

To determine whether mDC or pDC were facilitating latency in resting CD4^+^ T cells, we next co-cultured sorted mDC and pDC with SNARF-labelled resting CD4^+^ T cells for 24 hours prior to infection, and experiments were performed as described above. While productive infection was enhanced in both the mDC and pDC-T cell co-cultures (Fig. 2 A), latent infection was only identified in the SNARF^hi^EGFP^−^ CD4^+^ T cells that had been co-cultured with mDC (33 (19, 51) EGFP^+^ cells/10^4^ cells; n = 5) following re-stimulation with PHA and feeder PBMC (Fig. 2 B) or following direct activation with anti-CD3/CD28, together with IL-7 and the integrase inhibitor L8, which allowed the detection of post-integration latency (Fig. 2 C). To further confirm that post-integration latency was established in resting CD4^+^ T cells co-cultured with mDC, we used a real time PCR assay to quantify integrated HIV DNA. Integrated HIV DNA was present in SNARF^hi^EGFP^−^ CD4^+^ T cells sorted from the mDC co-cultures (1100 (686, 4960) HIV DNA copies/10^6^ cells, n = 3; Fig. 2 D), but not in the CD4^+^ T cells sorted from the pDC co-cultures or the CD4^+^ T cells cultured alone (both <330 HIV DNA copies/10^6^ cells). Similar results were observed with a nef competent EGFP-reporter virus (Fig. 2 E), demonstrating that the establishment of mDC-induced latency was not dependent on Nef.

Unlike experiments that utilised R5 EGFP HIV, when experiments were performed with an X4 EGFP reporter virus, latent infection was detected in the resting CD4^+^ T cells cultured alone (87 (51, 155) EGFP^+^ cells/10^4^ cells; n = 4; Fig. 2 F). However, latency was still significantly enhanced in the non-proliferating CD4^+^ T cells in the presence of mDC (468 (213, 621) EGFP^+^ cells/10^4^ cells) when compared to the T cells cultured alone.

In some experiments we added a low dose of Staphylococcus enterotoxin B (SEB; 10 ng/mL) to the mDC-T cell co-cultures to enhance productive infection and increase cognate interactions between mDC and T cells. In the presence of SEB we observed a significant increase in the level of productive infection; however, there was no difference in the level of latent infection (Fig. 2 G). Finally, we cultured mDC and T cells together at ratios ranging from 1∶10 to 1∶100 to determine the minimum interaction required between mDC and T cells to induce latency. We found that latency could still be established at a ratio of DC: T cells as low as 1∶100 (Fig. 2 H).

Taken together, these results demonstrated that *in vitro* mDC and not pDC facilitated post-integration latency in non-proliferating CD4^+^ T cells.

### Myeloid DC induce latency in memory CD4^+^ T cells

We have previously shown that memory CD4^+^ T cells and not naïve CD4^+^ T cells are susceptible to latent infection following chemokine exposure [14]. To determine whether mDC-induced T cell latency occurred in memory or naïve CD4^+^ T cells, we separated the SNARF^hi^EGFP^−^ CD4^+^ T cells into CD45RO^+^ (memory) and CD45RO^−^ (naïve) fractions prior to culture with feeder PBMC. In these experiments, latent infection was detected at significantly higher levels in the CD45RO^+^ memory CD4^+^ T cell fraction (146 (14, 197) EGFP+ cells/10^4^ cells; Fig. 2 I).

### Depletion of CD69 expressing cells has no effect on DC-induced latency

**Figure 3:**
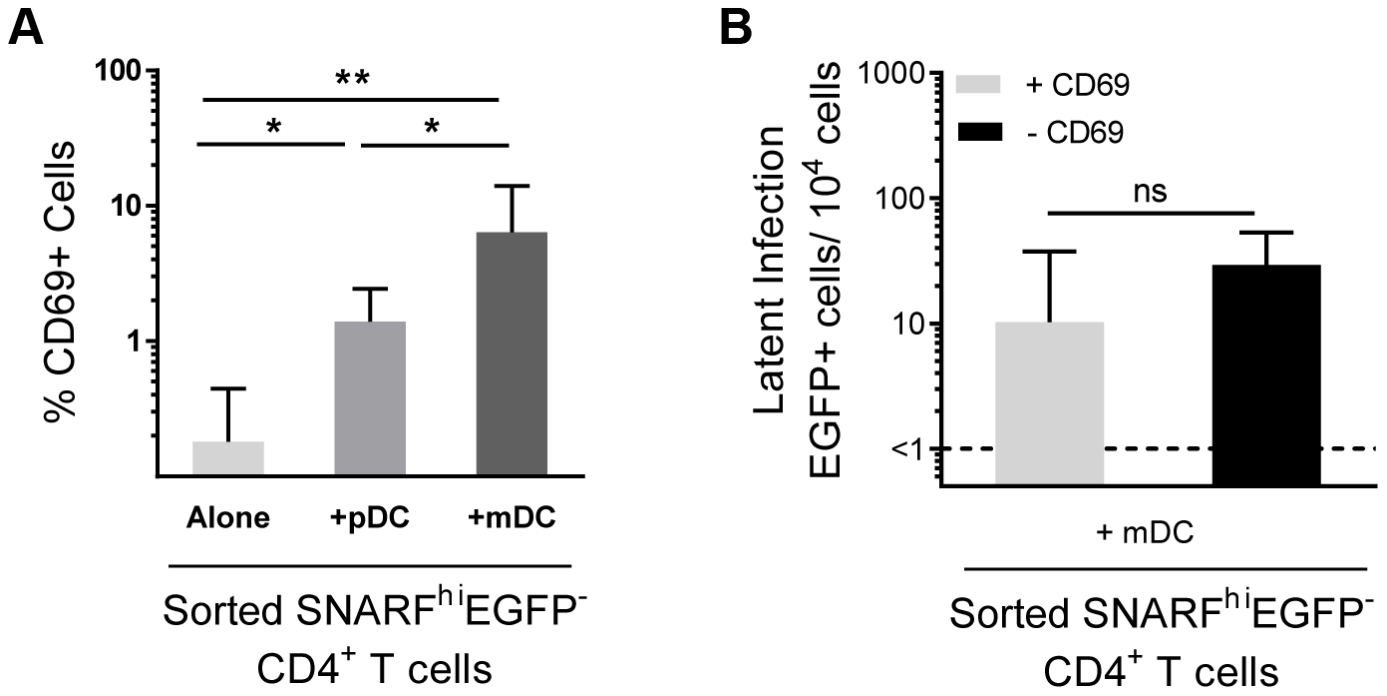
Depletion of CD69+ cells has no effect on mDC-induced latency. (**A**) Expression of CD69 was determined in sorted SNARF^hi^EGFP^−^ CD4^+^ T cells, n = 6. (**B**) Latent infection was determined for SNARF^hi^EGFP^−^ CD4^+^ T cells sorted from mDC-T cell co-cultures that either contained (+CD69, light grey) or were depleted of CD69-expressing cells (−CD69, black), n = 4. The lower limit of detection is represented by a dotted line. Columns represent the median of 4–6 donors and error bars indicate the interquartile range. *P<0.05; **P<0.01; ns, not significant (Wilcoxon signed-rank test).

A proportion of non-proliferating SNARF^hi^EGFP^−^ cells sorted from DC-T cell co-cultures (Fig. 1 F and 3 A) expressed CD69. Therefore, in order to exclude the possibility that we were only detecting infection of the cells showing early signs of activation, we depleted CD69^+^ cells from the SNARF^hi^EGFP^−^ T cells at day 5 post-infection prior to co-culture with PHA and feeder PBMC. We found no significant difference in the level of latency following depletion of the CD69^+^ cells (Fig. 3 B). CD69 expression can be transient, therefore, to confirm that we had not missed cells that expressed CD69, which had then been down-regulated; we measured the expression of CD69 over time following co-culture with mDC and infection with HIV. We demonstrated that CD69 expression peaked at day 2 and remained elevated out to day 5 post-infection (data not shown). These results demonstrate that the subpopulation of CD4^+^ T cells that were partially activated and expressing CD69 were not preferentially latently infected following mDC-T cell co-culture.

### HIV infection of mDC-T cell co-cultures leads to an increase in chemokine and cytokine expression

**Figure 4:**
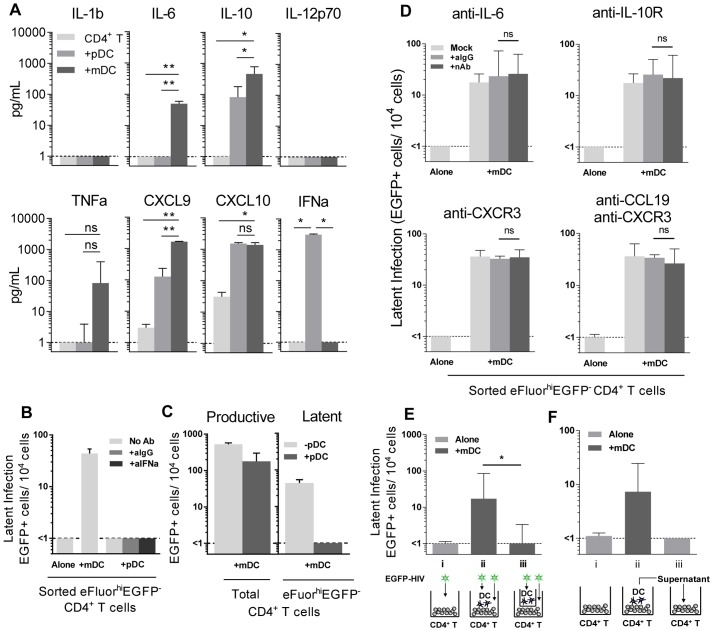
Soluble factors in DC-induced HIV latency. (**A**) Resting CD4^+^ T cells were cultured alone (light grey) or with sorted pDC (grey) or mDC (dark grey). At day 5 post-infection, cytokines and chemokines were quantified in culture supernatants using cytometric bead arrays, n = 3. *P<0.05; **P<0.01 (paired t-test). (**B**) Latent infection was quantified in eFluor670^hi^EGFP^−^ resting CD4^+^ T cells that were cultured either alone or with sorted pDC in the presence of media alone (light grey), anti-IgG (grey) or anti-IFN-alpha (dark grey) following stimulation with anti-CD3/CD28 in the presence of L8, n = 5. (**C**) Resting CD4^+^ T cells were co-cultured with mDC with (grey) or without (light grey) the addition of equal numbers of pDC. Productive infection was determined at day 5 post-infection. Latent infection was determined in sorted eFluor670^hi^EGFP^−^ CD4^+^ T cells following stimulation with anti-CD3/CD28 in the presence of L8, n = 5. (**D**) Latent infection was quantified in eFluor670^hi^EGFP^−^ resting memory CD4^+^ T cells that were cultured either alone or with sorted mDC in the presence of media alone (light grey), anti-IgG (grey) or neutralising antibodies (dark grey) to IL-6, IL-10-receptor, CXCR3 or CCL19, n = 5. (**E**) eFluor670-labelled resting CD4^+^ T cells were cultured either alone (light grey) or with blood mDC (dark grey). Virus was added to (**i**) CD4^+^ T cells cultured alone; (**ii**) CD4^+^ T cells co-cultured with mDC; (**iii**) CD4^+^ T cells cultured with mDC in the presence of a 0.4 µm membrane transwell and latency determined at day 5 post-infection, n = 5. (**F**) eFluor670-labelled resting CD4^+^ T cells were cultured either (**i**) alone (light grey) or (**ii**) with blood mDC (dark grey) and infected. (**iii**) Following 24 hours, supernatant from infected mDC-T cell co-cultures was added to uninfected resting CD4^+^ T cells and these cells were then infected, n = 3. Columns represent the median of 3–5 donors and error bars indicate the interquartile range. *P<0.05; **P<0.01 (Wilcoxon signed-rank test).

To determine why mDC and not pDC led to the establishment of latency, we compared cytokine levels in pDC-T cell and mDC-T cell co-cultures 5 days following HIV infection using bead arrays for known DC-secreted cytokines. Supernatants collected from HIV-infected mDC-T cell co-cultures compared to the HIV-infected pDC-T cell co-cultures had significantly increased expression of IL-6 (p = 0.002), IL-10 (p = 0.01) and CXCL9 (p = 0.002; Fig. 4 A). TNF-alpha was also up-regulated in the mDC-T cell compared to pDC-T cell co-cultures but the difference was not statistically significant (Fig. 4 A). As expected, IFN-alpha was detected at high levels in the pDC-T cell co-cultures but not in the mDC-T cell co-cultures (Fig. 4 A; p = 0.01), and latency was not established in pDC-T cell co-cultures even in the presence of neutralising antibodies to IFN-alpha (Fig. 4 B). Interestingly, when equal numbers of pDC and mDC were added to resting CD4^+^ T cells latency was reduced when compared to T cells cultured only with mDC (Fig. 4 C). This suggests that while pDC themselves do not induce latency, they are able to inhibit the establishment of latency mediated by mDC.

### Blocking Abs to IL-6, IL-10R, CXCR3 and CCL19 have no effect on DC-induced latency

To determine whether the soluble factors that were differentially expressed in mDC-T cell co-cultures compared to pDC-T cell co-cultures were contributing to the establishment of mDC-induced latency, neutralising antibodies (nAb), to either the soluble factor or its receptor, were added to the T cells prior to co-culture with DC and the addition of HIV. Specific nAbs or an anti-IgG control were added to eFluor670 (alternative proliferation dye to SNARF)-labelled resting memory (CD45RO^+^) CD4^+^ T cells prior to co-culture with mDC, and again following infection, and latency determined as described in Fig. 1 (Fig. 4 D).

When nAbs to IL-6, the IL-10 receptor (IL-10R) or CXCR3 (CXCL9 and CXCL10 receptor) were added to the mDC-T cell co-cultures, no significant decrease in the number of latently infected CD4^+^ T cells was observed when compared to cultures where control anti-IgG was added. As we had previously shown that the chemokine CCL19 can condition resting CD4^+^ T cells allowing for enhanced entry and integration of HIV [14], we also added anti-CCL19, either alone or in combination with anti-CXCR3, to the mDC-T cell co-cultures. However, we did not detect a significant decrease in the number of latently infected cells. The activity of these nAbs was confirmed by their ability to block STAT3 signalling (aIL-6, aIL-10R) or chemokine induced migration (aCXCR3, aCCL19; Fig. S2).

### DC-induced latency requires close proximity of DC and CD4^+^ T cells

To determine whether DC-T cell contact was required to establish latency in resting CD4^+^ T cells, we co-cultured mDC and resting CD4^+^ T cells separated by a 0.4 µm membrane transwell. Following 24 hours of culture, HIV was added to the mDC in the upper chamber and the CD4^+^ T cells in the lower chamber. Without mDC-T cell contact, the establishment of latency was significantly inhibited (<1 EGFP^+^ cell/10^4^ cells) when compared to co-cultures without membranes (18 (10, 85) EGFP^+^ cell/10^4^ cells; n = 5; p = 0.03; Fig. 4 E).

To further elucidate whether soluble factors other than those previously inhibited were involved in DC-induced latency, we added supernatant from infected mDC-T cell co-cultures to uninfected resting CD4^+^ T cells and then infected these cells with EGFP-HIV. Media changes were performed daily using supernatant from infected mDC-T cell co-cultures. Under these conditions the resting CD4^+^ T cells would be exposed to any soluble factors and free viral particles present in the mDC-T cell co-cultures but would not have any contact with the mDC. Latency was not detected in these cultures (Fig. 4 F), providing further evidence that DC-T cell contact, and not a soluble factor, was required for the establishment of DC-induced latency.

**Figure 5:**
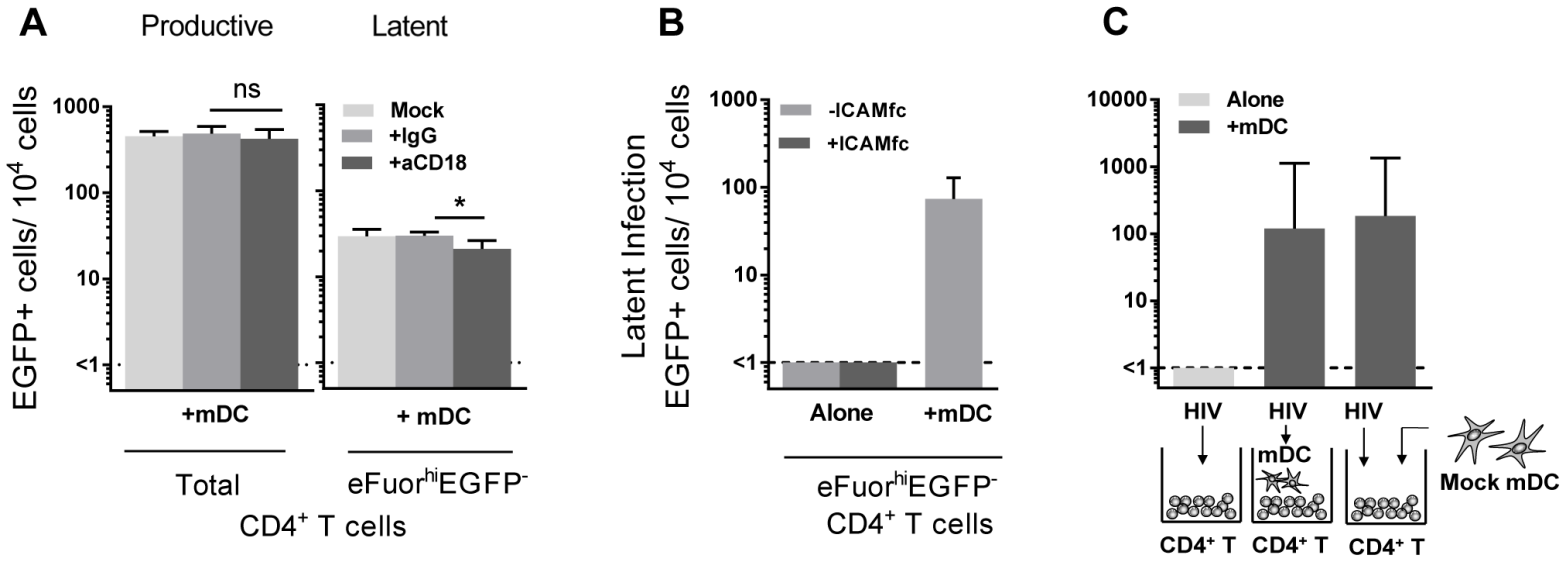
DC-T cell interactions. (**A**) Productive and latent infection was quantified in eFluor670-labelled resting memory CD4^+^ T cells that were cultured either alone or with sorted mDC in the presence of media alone (light grey), anti-IgG (grey) or anti-CD18 (dark grey) prior to infection, n = 5. (**B**) Latent infection was determined in eFluor670-labelled resting CD4^+^ T cells that were cultured alone, with mDC or alternatively with soluble ICAM-1-fc and anti-IgG-fc, n = 2. (**C**) Latent infection was determined in sorted eFluor^hi^EGFP^−^ CD4^+^ T cells following stimulation with anti-CD3/CD28, that were cultured alone or with mDC that were added prior to infection or post-infection, n = 5. Columns represent the median of 5 experiments and error bars the interquartile range. *P<0.05; ns, not significant (Wilcoxon signed-rank test).

Direct DC-T cell signalling can occur following interactions between several cell surface receptors. In particular, interactions between lymphocyte function associated antigen-1 (LFA-1; composed of CD11a and CD18) on T cells and intercellular adhesion molecule 1 (ICAM-1) on DC are involved in DC-T cell adhesion [22] and subsequent T cell activation via formation of an immunological synapse [23]. To inhibit DC-T cell clustering, we used blocking antibodies to CD18 (10–20 µg/mL). Blocking of CD18 significantly inhibited, but did not eliminate, DC-T cell clustering, as observed by microscopy (data not shown). Following incubation with anti-CD18, there was no effect on the number of productively infected cells, however, we observed a significant decrease in the number of latently infected cells from the mDC-T cell co-cultures (20 (8, 34) latently infected cells/10^4^ cells), when compared to cells cultured without anti-CD18 (25 (17, 48) latently infected cells/10^4^ cells; p = 0.03) or in the presence of control anti-IgG (32 (22, 36) latently infected cells/10^4^ cells; n = 6; p = 0.03; Fig. 5 A). However, when resting CD4^+^ T cells were stimulated with soluble ICAM-1 and anti-IgG there was no increase in latency observed (Fig. 5 B), suggesting that ICAM-LFA signalling alone does not induce latency in this model system. Furthermore, in the presence of anti-CD18 the number of latently infected cells from the mDC-T cell co-cultures remained greater than the CD4^+^ T cells cultured alone (<1 latently infected cell/10^4^ viable cells; p = 0.01) suggesting that the effect of anti-CD18 was most likely due to the partial decrease in clustering/DC-T cell contact.

In order to determine whether mDC transfer of HIV was involved in the establishment of latency we performed experiments where we added virus to resting CD4^+^ T cells, washed off virus and added uninfected mDC to the CD4^+^ T cells (Fig. 5 C). Under these conditions we were still able to detect latency in the non-proliferating CD4^+^ T cells. Together, these results indicate that cell-cell contact plays a role in DC-induced T cell latency but that the mDC were not required to be infected and then transfer HIV to the resting CD4^+^ T cells.

### Multiple genes are differentially expressed in latently infected, non-proliferating CD4^+^ T cells co-cultured with DC

**Figure 6:**
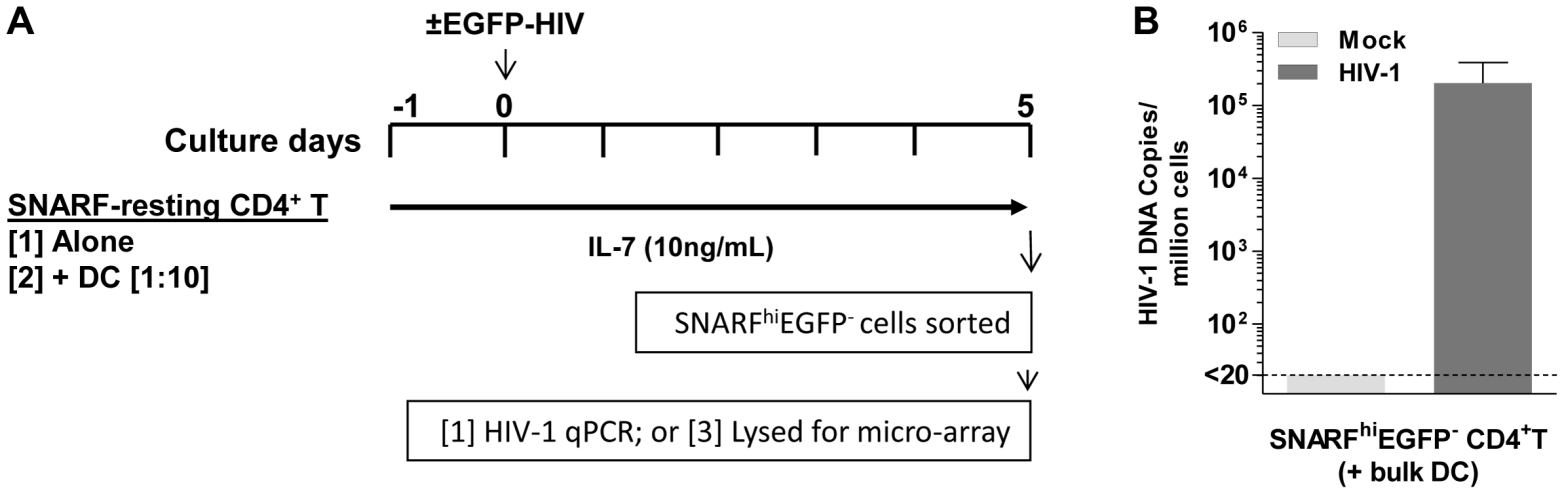
Acquisition of latently infected cells for microarray analysis. (**A**) SNARF-labelled resting CD4^+^ T cells were cultured with or without syngeneic blood DC. Following 24 hours of culture, cells were mock-infected with media alone (control) or infected with NL(AD8)-nef/EGFP (MOI of 5). Cells were cultured with IL-7 (10 ng/mL) throughout the culture period. On day 5 post-infection, SNARF^hi^EGFP^−^CD4^+^ T cells were sorted and lysed for either microarray or (**B**) qPCR, where total HIV DNA was quantified.

To determine the effect of DC on gene transcription in latently infected resting CD4^+^ T cells, SNARF-labelled resting CD4^+^ T cells, from four independent donors, were cultured either alone or with syngeneic bulk blood DC at a 1∶10 ratio for 24 hours prior to infection with NL(AD8)-nef/EGFP. In these experiments, we included IL-7 (10 ng/mL) in all cultures to increase cell survival of the resting cells and infections were performed at an MOI of 5 to ensure a high frequency of latently infected cells. Mock infections were performed in parallel with media alone. Non-proliferating (SNARF^hi^) CD4^+^ T cells that were not productively infected (EGFP^−^) were sorted 5 days post-infection and lysed for either the detection of HIV DNA by real-time PCR or RNA for microarray studies (Fig. 6 A).

Infection was confirmed in the resting CD4^+^ T cells following co-culture with DC, in 4 independent experiments, by detection of HIV DNA (3×10^4^ (7.4×10^3^, 5.7×10^5^) copies/10^6^ cells; Fig. 6 B). Changes in gene expression were quantified in the sorted SNARF^hi^EGFP^−^ CD4^+^ T cells using Illumina oligonucleotide microarrays. To identify genes expressed in DC-induced latency, we compared the expression profiles of non-proliferating, latently infected CD4^+^ T cells (HIV T (+DC)) to mock infected CD4^+^ T cells (Mock T (+DC)) that had been co-cultured with DC. In order to control for the effect of virus or DC alone, we first subtracted the gene expression profiles of control cells, which were T cells that had been cultured alone that were either uninfected (Mock T) or exposed only to virus (HIV T).

**Figure 7:**
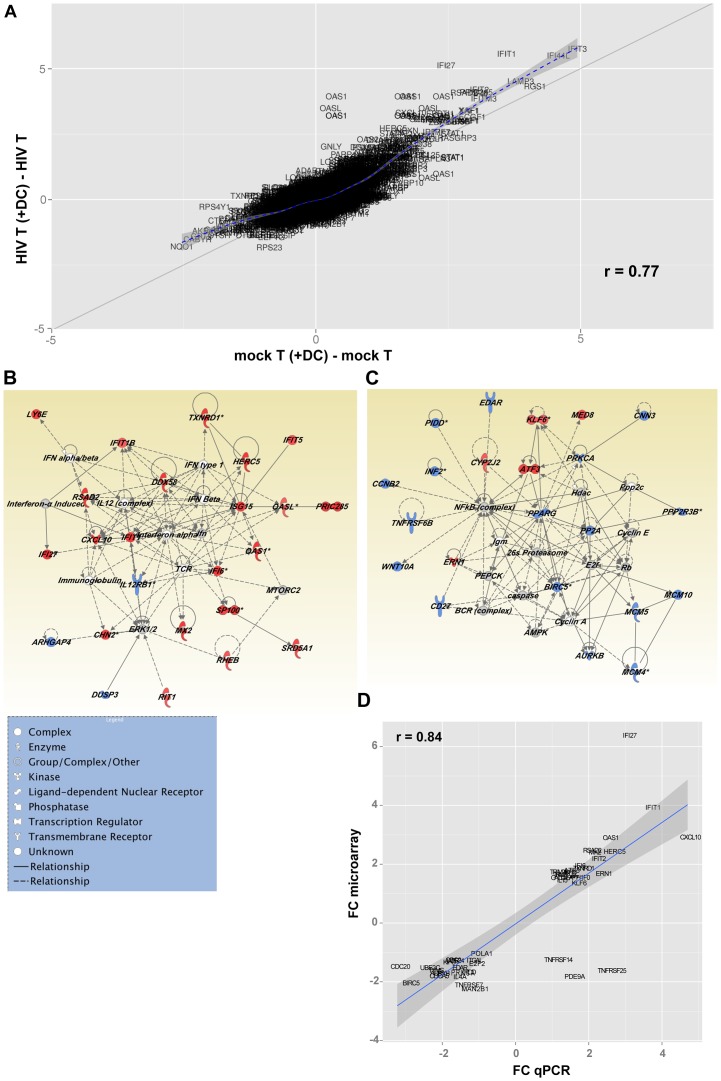
Changes in gene expression in DC-induced latently infected CD4^+^ T cells. (**A**) Fold change scatterplot comparing gene expression between HIV T (+DC) (CD4^+^ T cells cultured with DC and HIV), and Mock T (+DC) (CD4^+^ T cells cultured with only DC) relative to their controls, HIV T (CD4^+^ T cells cultured with HIV) and Mock T (CD4^+^ T cells cultured in media alone) respectively. The solid line indicates absolute 2-fold change. (**B** and **C**) Top two gene interaction networks as ranked by Ingenuity Pathway Analysis. The networks were built from the list of differentially expressed genes induced by HIV T (+DC), relative to Mock T (+DC) after subtracting HIV T and Mock T from each group respectively. Genes highlighted in red were up-regulated and those in blue were down-regulated. The different node shapes indicate genes in different functional categories according to the legend. The interactions between the different nodes are shown as solid (direct interaction) or dashed (indirect interaction) lines (edges). (**D**) Fold change in gene expression values for selected genes from the Illumina BeadArrays plotted against Real-Time PCR (qPCR) deltaCt values for each target gene. PCR targets were mapped to BeadArray probes by matching the official gene symbols.

A scatter plot (Fig. 7 A), representing the common (genes that fall on the diagonal) and differentially expressed genes (genes that fall off the diagonal) from this comparison, highlighted the significant differences in gene expression between latently infected cells and controls (r = 0.77). Additionally, this plot showed that several of the genes that discriminate latently infected cells from control cells were genes downstream of type I interferons, including interferon-induced protein with tetratricopeptide repeats 1 (IFIT-1), interferon alpha-inducible protein 27 (IFI27), and 2′-5′-oligoadenylate synthetase 1 (OAS1).

### Inhibition of cell cycle entry in latently infected, non-proliferating CD4^+^ T cells

Heatmaps of the top 100 genes (Fig. S3) confirmed the de novo induction of genes encompassing several biological and metabolic pathways in T cells exposed both to DC and virus. These included transcripts of the Interferon pathway, genes involved in the regulation of cell cycle entry and mitosis, as well as receptor and effector molecules of cell survival and apoptosis.

Network analysis (Fig. 7 B and C) showed that two major pathways were regulated in T cells following co-culture with DC exposed to HIV. Exposure of CD4^+^ T cells to HIV and DC led to the up-regulation of genes downstream of type I interferon by nucleotide sensors. Figure 7 B confirms the wide-ranging impact of the up-regulation of the type I Interferon pathway, as several molecules with antiviral activity were up-regulated, including ISG15 ubiquitin-like modifier (ISG15), and DEAD box polypeptide 58 (DDX58; also known as RIG-I). Genes involved in actin polymerisation, and the organisation of microtubules, were also up-regulated as a consequence of interferon pathway up-regulation. Additionally, the interferon pathway intercepted with the mammalian target of rapamycin complex 2 (mTORC2) pathway, which plays an important role in autophagy and T cell survival [24] (Fig. 7 B; Table S1).

Network analysis confirmed the negative impact of exposure of CD4^+^ T cells to DC and virus on the NF-κB pathway as well as several cellular metabolic pathways (fatty oxidation and glucose metabolism) regulated by peroxisome proliferator-activated receptor gamma (PPARG; Fig. 7 C). Inhibition of the NF-κB transcriptional network, which plays a significant role in HIV transcription, led to the down-regulation of protein kinase C alpha (PRKCA). This observation confirms the quiescence of these cells and may also be a step towards the induction of HIV latency. Triggering of a transcriptional program leading to T cell quiescence was confirmed by the increased expression of Kruppel-like factor 6 (KLF6), a gene with anti-proliferative functions [25], [26], as well as activating transcription factor 3 (ATF3) that has been recently been shown to negatively regulate activating protein 1 (AP-1)-mediated HIV transcription. Additionally, we observed a down-regulation of several molecules involved in DNA replication such as members of the minichromosome maintenance (MCM) complex (MCM4, MCM5, and MCM10) and the aurora kinase (Fig. 7 C). Pathways controlling pyrimidine and purine synthesis were also expressed at lower levels in cells exposed to virus and DC highlighting a reduced availability of nucleotides for cell division (Table S1). The down-regulation of NF-κB resulted in the decreased expression of several molecules that play a critical role in T cell survival, including CD27 (TNFRSF7), baculoviral IAP repeat containing 5 (BIRC5/survivin) and tumor necrosis factor receptor superfamily, member 6b (TNFRSF6B/DCR3), a decoy receptor that inhibits Fas ligand and LIGHT-mediated signalling [27]. In order to confirm the differential expression of genes in these different populations of cells, we used a highly quantitative PCR approach and showed a strong correlation between gene expression data measured by either gene array or PCR (Fig. 7 D and Table S2).

Taken together, results of transcriptional profiling highlighted the impact of two major transcriptional nodes in the inhibition of viral replication and the induction of latency. The up-regulation of Type I Interferons and their downstream target genes could trigger several genes endowed with antiviral activities and would also impact cell proliferation, survival and metabolic processes. Concomitantly, the down-regulation of NF-κB will lead to T cell quiescence and decreased levels of activation, both of which are required for HIV replication.

## Discussion

The study of latently infected resting CD4^+^ T cells *ex vivo* from HIV-infected patients on ART is greatly limited by the low frequency of latently infected cells and the lack of a distinctive surface marker to distinguish latently infected from uninfected cells. Here we demonstrate that latency can be efficiently established via direct infection of non-proliferating CD4^+^ T cells in the presence of DC. Using primary blood DC and resting CD4^+^ T cells we have demonstrated that: [1] co-culture of resting memory CD4^+^ T cells with DC can establish latent infection; [2] mDC but not pDC mediate this effect; [3] close cell-cell proximity is required between DC and T cells; and [4] multiple cell cycle genes were altered in non-proliferating CD4^+^ T cells, containing latently infected cells. These novel findings provide a potential pathway for the establishment and maintenance of latent infection in resting CD4^+^ T cells that recapitulates the likely events within lymphoid tissues in HIV-infected patients *in vivo*.

Previous studies have explored the ability of DC to enhance productive HIV infection within DC-CD4^+^ T cell co-cultures [28]–[31]; however, we are the first to present data demonstrating the ability of specific subpopulations of DC to induce latency in resting CD4^+^ T cells in these co-cultures. Using this model we clearly demonstrated that following co-culture of mDC with resting memory CD4^+^ T cells, post-integration latency was established. This was demonstrated by inducible virus (established both in the presence and absence of indinavir) and detectable integrated HIV DNA in T cells cultured with mDC but not those cultured alone following infection with an R5 EGFP reporter virus. While integrated R5 HIV DNA was only detected following co-culture with mDC in our model of latency, it was similar to that previously reported for resting CD4^+^ T cells infected in isolation with a wild type X4 NL4.3 virus [32]. Resting CD4^+^ T cells express very low levels of CCR5 in contrast to expressing very high levels of CXCR4. Additionally, we saw significantly higher levels of latency (∼100 fold) in our T cells cultured alone when we used an X4 EGFP reporter virus compared to an R5 EGFP virus.

Unlike memory CD4^+^ T cells, we were unable to detect latency in naïve CD4^+^ T cells following mDC co-culture. It is possible that the differential establishment of latency in resting naïve and memory T cells was due to differences in their cortical actin density and actin dynamics as previously suggested by others [33]. While mDC induced T cell latency in this model, pDC did not. Interestingly, pDC played an active inhibitory role in establishing latency, when co-cultures were performed with equal numbers of pDC and mDC (Fig. 4 C). One potential explanation for the difference between co-cultures of bulk DC and T cells (where latency was established) and DC-T cell co-cultures containing purified equal numbers of pDC and mDC (where latency was inhibited) could potentially be that the number of pDC present in the bulk DC (roughly 1 pDC to 3 mDC) was too low to inhibit latency. How pDC actively suppress the establishment of latency is unknown, but it does not appear to be mediated by IFN-alpha.

Establishment of mDC-induced latency was not dependent on DC-T cell transfer of HIV, as latency was still detected when T cells were infected in isolation and uninfected mDC added only after virus had been washed off. Nor was it dependent on the amount of virus replication, because while only mDC were able to induce latent infection, similar levels of productive infection were observed in both the pDC and mDC co-cultured CD4^+^ T cells (Fig. 2 A). Furthermore, we found that addition of SEB to the culture model enhanced productive infection but did not increase latent infection (Fig. 2 G). Together, these data provide evidence that the establishment of latency in the non-proliferating CD4^+^ T cells when co-cultured with mDC was not simply due to higher viral exposure in these cultures.

These results differ from a previous study that also looked at direct infection of resting CD4^+^ T cells, which concluded that DC had no effect on the integration levels of R5 or X4 virus in either naïve or memory CD4^+^ T cells [34]. However, in this study, although primary DC were used (defined as BDCA-1^+^ and BDCA-4^+^ cells), total DC were present at a frequency of only 0.89% and therefore the frequency of mDC may have been too low to demonstrate an effect of mDC on the infection of resting CD4^+^ T cells. Additionally, contrary to our data, a recent study has reported the ability of monocyte derived DC (MDDC) to activate latent infection in T cells [35]. A key difference was that latency in this study was unusually established in proliferating CD4^+^ T cells and not non-proliferating T cells as in our study. Furthermore, MDDC, as opposed to primary DC, were utilised in this study. MDDC have multiple significant functional and lineage differences to primary DC as we have recently demonstrated using detailed sorting and gene expression analyses [36].

In this study we utilised total blood CD11c^+^ mDC, which consist of at least three different subsets, a major SLAN (6-sulfo LacNAc^+^), an intermediate CD1c^+^ (BDCA-1) and a minor CD141^+^ (BDCA-3) population, each with different functional properties [37]–[40]. However, it is currently unclear whether one or more of these subsets is responsible for inducing latency in resting CD4^+^ T cells.

We have previously demonstrated that multiple chemokines, including CCL19, CXCL9 and CXCL10, can condition resting CD4^+^ T cells allowing for the establishment of HIV latency [14], [17]. However, blocking CCL19 and CXCR3, the receptor for CXCL9, 10 and 11, had a minimal impact on DC-induced latency (Fig. 4 D). While it is possible that there may be involvement of chemokines other than those inhibited, given that latency was not detected in resting CD4^+^ T cells infected in the presence of infected mDC-T cell culture supernatants, this is unlikely (Fig. 4 F). Rather, our data supports an essential role for direct DC-T cell interactions or DC-T cell signalling as mDC-induced latency was prevented when the mDC were cultured in transwells above the resting CD4^+^ T cells (Fig. 4 E). Unlike our previous work that was performed in the absence of productive infection [14], latency following DC-T cell co-culture was established in the presence of productive infection, which may more accurately mimic the establishment of latency in acute infection *in vivo*. Therefore, in the presence of productive infection it is possible that there are alternative pathways that lead to the establishment and maintenance of latency in resting CD4^+^ T cells.

Interactions between ICAM-1, found on DC, and LFA-1, found on T cells, strengthen DC-T cell adhesion and play a key role in the formation of the immunological synapse [41]. We have shown that clustering, facilitated by ICAM-1-LFA-1 interactions, contributed to DC-induced T cell latency, as latency was significantly reduced, but not eliminated, when blocking antibodies to CD18/LFA-1 were added to the DC-T cell co-cultures (Fig. 5 A). However, interactions between ICAM-1 and LFA-1 alone were not sufficient to induce T cell latency in the absence of mDC (Fig. 5 B), therefore, the reduction in latency observed in the presence of anti-CD18 was most likely due to the reduction in DC-T cell clustering rather than specific LFA-ICAM signalling events. As there are numerous other molecules involved in DC-T cell clustering, such as LFA-3 and CD2, additional signalling pathways should be explored as potential mediators of DC-induced HIV latency. Interestingly, a recent paper has demonstrated enrichment of latency in CD2 expressing T cells from HIV-infected patients on ART [42].

Transcriptional profiling experiments served to highlight changes in cellular gene expression in resting non-proliferating CD4^+^ T cells that contained latently infected CD4^+^ T cells. We showed significant differences in gene expression between resting CD4^+^ T cells from HIV and mock infected DC-T cell cultures. However, it is important to note that, while all cells within our “latent” cell population were exposed to virus, only a proportion were actually infected (median of 3%). It is possible that some of the observed differences in gene expression may be due to uninfected cells that were exposed to HIV but not infected. This may include the genes downstream of type I interferons as our *in vitro* experiments have shown that pDC, the major producers of type I interferons, were not involved in the induction of T cell latency. Therefore, it is possible that type I interferons are necessary but alone are not sufficient to induce HIV latency. We have demonstrated significant differential expression of genes involved in cell cycle, in particular those associated with cell cycle arrest (Fig. 7 C). During DC-T cell interactions in the presence of HIV, differential expression of co-stimulatory and negative regulatory factors determines the fate of the interacting CD4^+^ T cell [43]. These interactions can result in active suppression of T cell cycle and as a result may inhibit post-integration steps in viral replication and promote the establishment of latency. Indeed, in HIV-infected patients on ART, HIV DNA is found at higher frequencies in CD4^+^ T cells expressing the negative regulator PD-1 [44].

Latency has also been shown to be triggered by the absence of certain transcriptional machinery in resting CD4^+^ T cells, such as NF-κB [45] and nuclear factor of activated T (NFAT) [11], [46]. In DC-induced latently infected CD4^+^ T cells we observed the suppression of multiple genes associated with the activation of NF-κB (Table S1), including protein kinase C alpha, PRKCA, which also plays a role in the activation of NFAT [47], [48]. Therefore, it is possible that the global suppression of genes associated with the activation of NF-κB and/or NFAT may also contribute to the maintenance of latency in DC- T cell co-cultures by preventing progression to productive infection in cells that contain integrated HIV. However, while this data provides insights into genes that may potentially be important for both the establishment and maintenance of latency, it will be important to conduct gene knockdown experiments within our model in order to determine the specific role of individual genes in establishing and maintaining mDC-induced T cell latency.

In summary, this study has demonstrated a novel pathway for the establishment of latency in resting memory CD4^+^ T cells that was dependent on close proximity to mDC. Efficient infection of resting CD4^+^ T cells in close contact with mDC and HIV could explain the rapid early establishment of the latent HIV reservoir. Additionally, if infectious virus persists in tissues such as lymph node in patients on ART, mDC may facilitate ongoing infection of resting T cells leading to replenishment of the reservoir.

## Materials and Methods

### Isolation of cellular subsets

PBMC were isolated from buffy coats obtained from the Australian Red Cross Blood Service (Melbourne, Australia). Resting CD4^+^ T cells were negatively selected using magnetic cell sorting and a cocktail of antibodies to CD8, CD11b, CD16, HLA-DR, CD19 and CD69, as previously described [17], [49]. Sorted cells were routinely negative for CD69, CD25 and HLA-DR (Fig. S1 A). In some experiments bulk resting CD4^+^ T cells were further sorted into CD45RA^+^ naïve and CD45RA^−^ memory CD4^+^ T cells using phycoerythrin (PE)-labelled antibody to CD45RA and a FACSAria (BD Biosciences). DC were isolated from blood as previously described [50]. Briefly, DC were enriched using magnetic bead depletion and antibodies to CD3, CD11b and CD19. Enriched cells were then sorted using a FACSAria (BD Biosciences) to obtain a bulk cocktail^−^ HLA-DR^+^ DC population, HLA-DR^+^CD11c^+^ mDC or HLA-DR^+^CD123^+^ pDC. The purity of sorted cells was always >98% (Fig. S1 B).

### Plasmids, virus production and infection

In all experiments except where noted we used an NL4-3 virus with EGFP inserted into the *nef* open reading frame at amino acid position 75 at the aKpnI (Acc651) site with a CCR5-tropic (AD8) envelope (NL(AD8)-nef/EGFP), alternatively we used this virus with a CXCR4-tropic (NL4-3) envelope (NL4-3-nef/EGFP; both kindly provided by Damian Purcell, University of Melbourne, Melbourne, Australia). In one set of experiments we used a Nef-competent EGFP reporter virus, kindly provided by Yasuko Tsunetsugu-Yokota (National Institute of Infectious Diseases, Tokyo, Japan) [51]. HIV stocks were generated by FuGene (Promega, Madison, WI) transfection of 293T cells as previously described [49], [50]. Cells were infected at 37°C for 2 hours at an MOI of 0.5 or 5, as determined by limiting dilution using the Reed and Muench method [52], followed by a wash step to remove unbound virus.

### In vitro HIV latency model

Resting CD4^+^ T cells were labelled with proliferation dye, either SNARF (10 µM; Invitrogen) or eFluor®670 (5 µM; eBiosciences, San Diego, CA), according to the manufacturer's instructions. SNARF/eFluor670-labelled resting CD4^+^ T cells were cultured in media supplemented with IL-2 (2 U/mL; Roche Diagnostics) for 24 hours, with or without syngeneic bulk DC or sorted DC subsets (DC: T cell ratio of 1∶10), in the presence or absence of SEB (10 ng/mL; Sigma). Cells were then infected using an EGFP-reporter virus and cultured for a further 5 days (Fig. 1 A). In some experiments, cells were cultured with and without the protease inhibitor Indinavir (0.1 µM final) for 30 minutes at 37°C prior to infection. At day 5 post-infection, cells were analysed by flow cytometry for productive infection by detecting EGFP^+^ cells. Subsequently, the non-proliferating (SNARF^hi^/eFluor670^hi^) CD4^+^ T cells that were not productively infected (EGFP^−^) were sorted using a FACSAria (BD Biosciences). In order to amplify any latent infection, the sorted CD4^+^ T cells were stimulated with PHA (10 ug/mL)/IL-2 (10 U/mL) in the presence of PBMC and cultured for a further 5 days. The number of EGFP^+^ cells following re-stimulation was used as a surrogate measure for the number of latently infected, non-proliferating CD4^+^ T cells in the original cultures (Fig. 1 B). In some experiments, we also stimulated the sorted SNARF^hi^/eFluor670^hi^EGFP^−^ CD4^+^ T cells directly with plate bound anti-CD3 (Beckman Coulter; 5 µg/mL) and soluble anti-CD28 (BD; 5 µg/mL). Flat bottomed 96 well plates were coated with anti-CD3 (5 µg/mL) for 3 hours at 37°C. Unbound antibody was then removed and 1×10^5^ SNARF^hi^/eFluor670^hi^EGFP^−^ CD4^+^ T cells were plated per well in 200 µL of media containing soluble anti-CD28 (5 µg/mL), IL-7 (50 ng/mL) and the integrase inhibitor, L8 (1 µM final). The number of EGFP^+^ cells was determined following 72 hours of culture. As a control for the activity of the integrase inhibitor L8, we cultured SEB-stimulated PBMC with and without L8 for 30 minutes prior to infection, and productive infection was determined at day 5 post-infection (Fig. S2).

### Phenotyping

For phenotypic analysis of the CD4^+^ T cells before culture, we stained sorted resting CD4^+^ T cells with CD69-FITC, CD25-PE and HLA-DR-perCP (BD Bioscience) on ice for 25 minutes. To determine whether co-culture with DC had altered the activation state of the resting CD4^+^ T cells, in some experiments the sorted SNARF^hi^EGFP^−^ CD4^+^ T cells were labelled with either CD69-FITC or HLA-DR-FITC (BD Biosciences). Intracellular staining was also performed on the sorted SNARF^hi^EGFP^−^ CD4^+^ T cells to detect expression of the cell cycle marker Ki67. Cells were permeabilised with 500 µL of 1× FACS Permeabilising Buffer (BD Biosciences) in the dark at room temperature for 10 minutes, washed once with FACS wash and incubated with Ki67-FITC (5 µL/10^5^ sorted CD4^+^ T cells; Dako) for 45 minutes on ice. Following incubation, cells were washed twice with FACS wash and resuspended in 1% FACS fix. We performed analyses on a FACSCalibur (BD Biosciences) and results were analysed using Weasel software (Walter and Elisa Hall Institute, Melbourne, Australia).

### Bead arrays

Cytokine bead arrays (eBioscience) were used according to the manufacturer's directions to determine the concentration of IL-1-beta, IL-6, IL-10, IL-12p70, TNF-alpha, CXCL9 and CXCL10 in the cell cultures.

### Inhibition of soluble factors/surface receptors

In some experiments, nAbs to CD18 (10 µg/mL (prior to infection) and 20 µg/mL (post-infection); clone 7E4; Beckman Coulter), CCL19 (25 µg/mL), CXCR3 (20 µg/ml), IFN-alpha (5 µg/mL) or control IgG (R&D Systems, Minneapolis, MN); IL-6 or IgG1 (10 ug/mL; BioLegend, San Diego, CA); IL-10R or IgG2 (10 ug/mL; Biolegend) were used. In these experiments, both the DC and the resting CD4^+^ T cells were pre-incubated with nAbs for 15 minutes on ice prior to culture. The nAbs were added again to the co-cultures following infection. Neutralising activity of anti-CCL19 (25 µg/mL) and anti-CXCR3 (20 µg/ml) was confirmed using a chemokine-induced migration assay. Resting CD4^+^ T cells were added to the top chamber of a 3 µM pore transwell migration plate (Sigma) and either CCL19 (100 nM) or CXCL10 (300 nM) was added to the bottom chamber. In experiments using anti-CXCR3, cells were treated with nAb for 15 minutes at 37°C and washed off prior to chemokine treatment. In comparison, in experiments using anti-CCL19, nAb was added together with chemokine to the bottom chamber. Migrated cells in the bottom chamber were then counted in duplicate at 20 hours post addition of chemokine. Anti-IL-6 and anti-IL-10R were used at neutralising concentrations previously described [53], [54]. As positive controls for these nAbs we demonstrated that 10 µg/mL of anti-IL-6 and anti-IL10R or 5 µg/mL of anti-IFN-alpha efficiently blocked IL-6 (100 ng/mL), IL-10 (50 ng/mL) or IFN-alpha (50 ng/mL) mediated STAT3 phosphorylation respectively (Fig. S2).

### ICAM-fc stimulation

In order to determine the role of ICAM-1 and LFA-1 interactions in mDC-induced latency, resting CD4^+^ T cells were cultured alone or with 10 ug/mL of ICAM-1fc together with 6 µg/mL of anti-IgG-fc (both from R&D Systems) for 24 hours prior to infection and maintained post-infection.

### Transwell/transfer experiments

DC were cultured with resting CD4^+^ T cells in the presence and absence of 0.4 µm cell culture inserts (BD, Franklin Lakes, NJ) with DC in the top chamber and resting CD4^+^ T cells in the lower chamber. Following 24 hours of culture, both the DC and the CD4^+^ T cells were infected as described above. In other experiments, we added supernatant from infected mDC-T cell co-cultures to uninfected resting CD4^+^ T cells and then infected these cells. Media changes were performed daily using supernatant from infected mDC-T cell co-cultures. To determine the role of DC-T cell transfer, resting CD4^+^ T cells were infected in the absence of mDC and uninfected mDC were added back to the T cells only after virus had been washed off.

### Microarrays

SNARF^hi^EGFP^−^ CD4^+^ T cells cultured with DC, in the presence (latently infected) or absence (mock-infected) of HIV, were sorted 5 days following infection with NL(AD8)-nef/EGFP. In these experiments, all culture media was supplemented with 10 ng/mL of IL-7 (Sigma) instead of IL-2, in order to increase cell survival of resting cells, and infections were performed at an MOI of 5 to ensure high numbers of latently infected cells.

Microarrays were performed as previously described [55]. Briefly, cells were lysed and RNA extracted (Qiagen, Valencia, CA), amplified (Ambion Applied Biosystems, Austin, TX) and hybridised to an Illumina Human-Ref8 (v3) BeadChip (Illumina, San Diego, CA). Beadchips were scanned using an Illumina BeadStation 500GX scanner and Illumina BeadStudio (version 3) software (Ilumina). Illumina probe data was exported from BeadStudio as raw data and screened for quality. Samples failing chip visual inspection and control examination were removed. Gene expression data was analysed using Bioconductor (http://bioconductor.org/) [56], an open-source software library for the analyses of genomic data based on R, a language and environment for statistical computing and graphics (www.r-project.org). The R software package was used for pre-processing, first to filter out genes with intensities below background in all samples, then to minimum-replace (a surrogate-replacement policy) values below background using the mean background value of the built-in Illumina probe controls as an alternative to background subtraction (which may introduce negative values) to reduce “over inflated” expression ratios determined in subsequent steps, and finally quantile-normalise the gene probes intensities. Genes were then filtered by intensity and by variance filters to allow a reduction in the number of tests and a corresponding increase in power of the differential gene expression analysis. The resulting matrix showing filtered genes as rows and samples as columns was log_2_ transformed and used as input for linear modelling using Bioconductor's *limma* package, which estimates the fold-change between two predefined groups by fitting a linear model and using an empirical Bayes method to moderate standard errors of the estimated log-fold changes for expression values from each gene. *P* values from the resulting comparison were adjusted for multiple testing according to the method of Benjamini and Hochberg [57]. This method controls the false discovery rate, which was set to 0.05 in this analysis. Microarray data is available through the National Center for Biotechnology Information Gene Expression Omnibus (GEO), series accession number pending.

### Functional microarray analysis and network generation

Ingenuity Pathway Analysis (IPA) software (Ingenuity Systems, www.ingenuity.com) was used to identify canonical signalling pathways and networks associated with the expression profiles of the non-proliferating CD4^+^ T cells cultured with DC in the presence (HIV T (+DC)) or absence (Mock T (+DC)) of HIV. Differentially expressed Illumina Probe IDs were imported into the Ingenuity software and mapped to the Gene Symbol from Ingenuity knowledge database. The significance of the association between the dataset and the canonical pathway was measured in two ways: 1) A ratio of the number of genes from the dataset that map to the pathway divided by the total number of genes that map to the canonical pathway; 2) Over-representation Fisher's exact test was used to calculate a p-value determining the probability that the association between the genes in the dataset and the canonical pathway is explained by chance alone. The pathways were ranked by −log p-value. This score was used as the cut-off for identifying significant canonical pathways (p value<0.05). IPA's networks are built from direct or indirect physical, transcriptional, and enzymatic interactions between the mapped genes (focus genes). Two genes are considered to be connected if there is a path in the network between them. Ingenuity's approach is based on a multi-stage, heuristic algorithm that iteratively constructs networks that greedily optimize for both interconnectivity and number of Focus Genes under the constraint of a maximal network size. Each individual IPA network has a maximum of 35 focus genes and is assigned a significance score (based on *P* value) representing the likelihood that the focus genes within the network are found there by random chance.

### Real-time PCR analysis

As previously described, full length viral DNA was quantified using primers specific for the HIV-1 long terminal repeat (LTR) and Gag [58], and integrated HIV-1 DNA was quantified using a nested Alu-LTR real-time PCR [15], [59], [60]. Results were normalised for total input DNA as determined by real-time PCR for the CCR5 gene [61]. The correlation between gene arrays and real-time PCR was performed using a Spearman correlation test.

Microarray expression data were validated in two donors by reverse transcriptase real-time PCR (RT-qPCR), as previously described [62]. Briefly, SNARF^hi^EGFP^−^ CD4^+^ T cells were lysed for RNA extraction and DNAse treatment (Qiagen, RNAeasy mini kit). cDNA was generated using CellsDirect qRT-PCR mix (Invitrogen). After reverse transcription all target genes were pre-amplified (18 cycles) using Taqman primers (Roche Probe library) specific for the transcripts of interest, which were also used for quantification. qPCR were performed on a Roche Light Cycler 348II and analysed according to the ΔΔct method.

### Statistical analysis

In all experiments, Wilcoxon signed-rank or student paired t tests (for n<5) were performed for comparisons between populations using Graphpad Prism 5.0 software. *P* values of less than 0.05 were considered significant. Statistical analyses for microarray data were performed with program R, according to the method of Benjamini and Hochberg [57].

## Supporting Information

Figure S1 **Gating strategy and phenotype of isolated cell populations.** (**A**) Resting CD4^+^ T cells were isolated from PBMC from the blood of healthy donors using antibodies to CD8, CD11b, CD14, CD16, CD19, CD69 and HLA DR and magnetic bead depletion. Purity was always greater than 98% and the sorted CD4^+^ T cells were negative for the activation markers CD69, CD25 and HLA-DR. (**B**) Syngeneic blood dendritic cells (DC) were enriched using magnetic bead depletion and antibodies to CD3, CD11b and CD19. Enriched cells were then sorted using a FACSAria into an HLA-DR^+^ DC population or further sorted into HLA-DR^+^CD11c^+^ myeloid DC (mDC) or HLA-DR^+^CD123^+^ plasmacytoid DC (pDC). The purity of sorted cells was always >98%. (**C**) Phenotypic analysis of sorted pDC and mDC before culture.
(PDF)

Figure S2 **Drug and nAb controls.** (**A**) SEB-stimulated PBMC were cultured with or without 1 µM L8 for 30 minutes prior to infection with NL(AD8)-nef/EGFP. Productive infection (EGFP^+^ cells) was determined at day 5 post-infection. (**B**) Neutralising activity of anti-CCL19 (25 µg/mL) was confirmed using a chemokine-induced migration assay. (**C**) Neutralising activity of anti-IL-10R (10 µg/mL), anti-IL-6 (10 µg/ml) and anti-IFN-alpha (5 µg/mL) was confirmed by their ability to efficiently blocked IL-6 (100 ng/mL), IL-10 (50 ng/mL) or IFN-alpha (50 ng/mL) mediated STAT3 phosphorylation respectively.
(PDF)

Figure S3 **Top differentially expressed genes.** Supervised clustering heatmap of the top differentially expressed genes resulting from comparing HIV T (+DC) and Mock T (+DC) samples after subtracting HIV T (CD4^+^ T cells cultured with HIV) and Mock T (CD4^+^ T cells cultured in media alone) from each group respectively. Genes were selected as differentially expressed based on Fold Change (≥1.5 fold up or down-regulation) and a p-value<0.05, following a moderate t test as implemented in the LIMMA package. The scale shows the level of gene expression where red and blue correspond to up and down-regulation respectively.
(PDF)

Table S1 **Significant pathways.** Significant pathways differentially expressed in HIV (+DC) relative to Mock T (+DC) after the subtraction of HIV T and Mock T respectively. Gene symbols are colour coded indicating either up-regulation (red) or down-regulation (blue). © 2000–2013 Ingenuity Systems, Inc. All rights reserved.
(PDF)

Table S2 **RT-PCR validated genes.** Fold change obtained from either gene-array or RT-PCR representing the change in expression level for each gene in HIV T (+DC) relative to Mock T (+DC) after the subtraction of HIV T and Mock T respectively.
(PDF)
