# Correction: Myeloid Dendritic Cells Induce HIV-1 Latency in Non-proliferating CD4^+^ T Cells

**DOI:** 10.1371/journal.ppat.1004486

**Published:** 2014-10-02

**Authors:** The PLOS Pathogens Staff

In the Materials and Methods section, in the last sentence of the subsection titled “Microarrays”, there is a missing series accession number. Instead of “series accession number pending,” it should read “series accession number GSE52344.”
