# Supplementary figures and images for: Myeloid Dendritic Cells Induce HIV-1 Latency in Non-proliferating CD4+ T Cells
Source: PLoS Pathog. 2013 Dec 5;9(12):e1003799. doi: 10.1371/journal.ppat.1003799 (PMC3855553; doi:10.1371/journal.ppat.1003799)

Figure S1

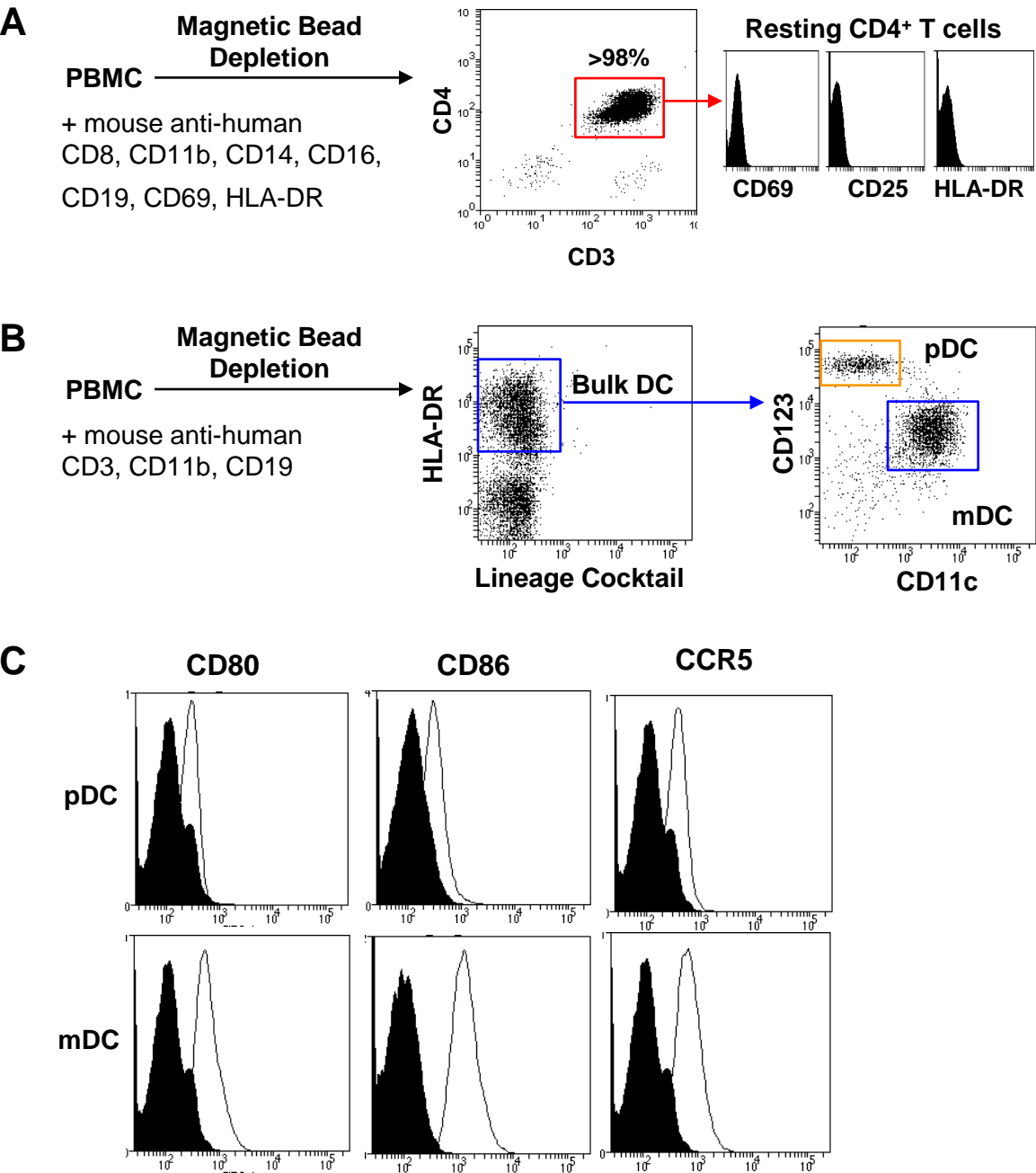

Supplement: Figure S1 — Gating strategy and phenotype of isolated cell populations. (A) Resting CD4+ T cells were isolated from PBMC from the blood of healthy donors using antibodies to CD8, CD11b, CD14, CD16, CD19, CD69 and HLA DR and magnetic bead depletion. Purity was always greater than 98% and the sorted CD4+ T cells were negative for the activation markers CD69, CD25 and HLA-DR. (B) Syngeneic blood dendritic cells (DC) were enriched using magnetic bead depletion and antibodies to CD3, CD11b and CD19. Enriched cells were then sorted using a FACSAria into an HLA-DR+ DC population or further sorted into HLA-DR+CD11c+ myeloid DC (mDC) or HLA-DR+CD123+ plasmacytoid DC (pDC). The purity of sorted cells was always >98%. (C) Phenotypic analysis of sorted pDC and mDC before culture. (PDF) [file ppat.1003799.s001.pdf]

# Figure S2

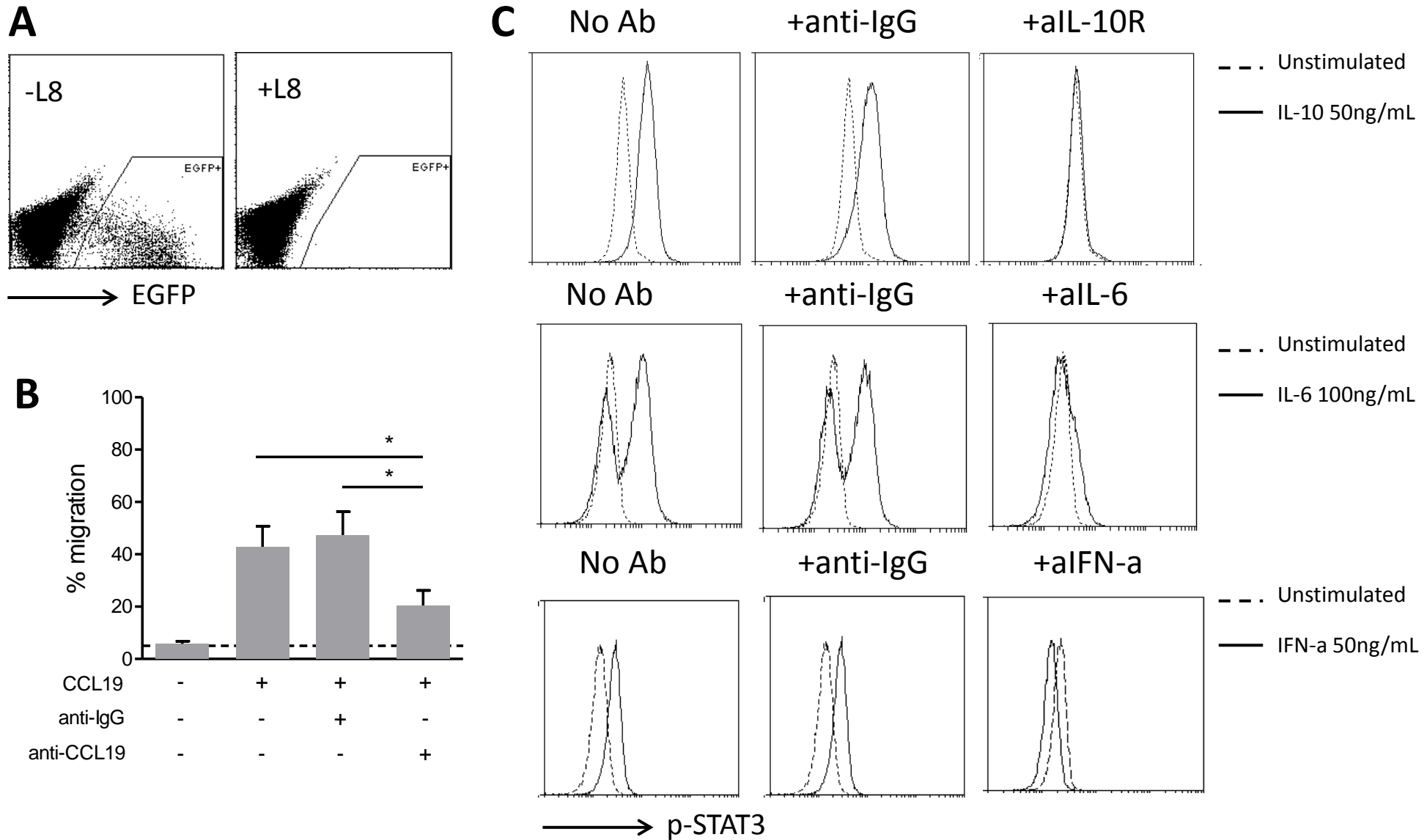

Supplement: Figure S2 — Drug and nAb controls. (A) SEB-stimulated PBMC were cultured with or without 1 µM L8 for 30 minutes prior to infection with NL(AD8)-nef/EGFP. Productive infection (EGFP+ cells) was determined at day 5 post-infection. (B) Neutralising activity of anti-CCL19 (25 µg/mL) was confirmed using a chemokine-induced migration assay. (C) Neutralising activity of anti-IL-10R (10 µg/mL), anti-IL-6 (10 µg/ml) and anti-IFN-alpha (5 µg/mL) was confirmed by their ability to efficiently blocked IL-6 (100 ng/mL), IL-10 (50 ng/mL) or IFN-alpha (50 ng/mL) mediated STAT3 phosphorylation respectively. (PDF) [file ppat.1003799.s002.pdf]

Figure S3

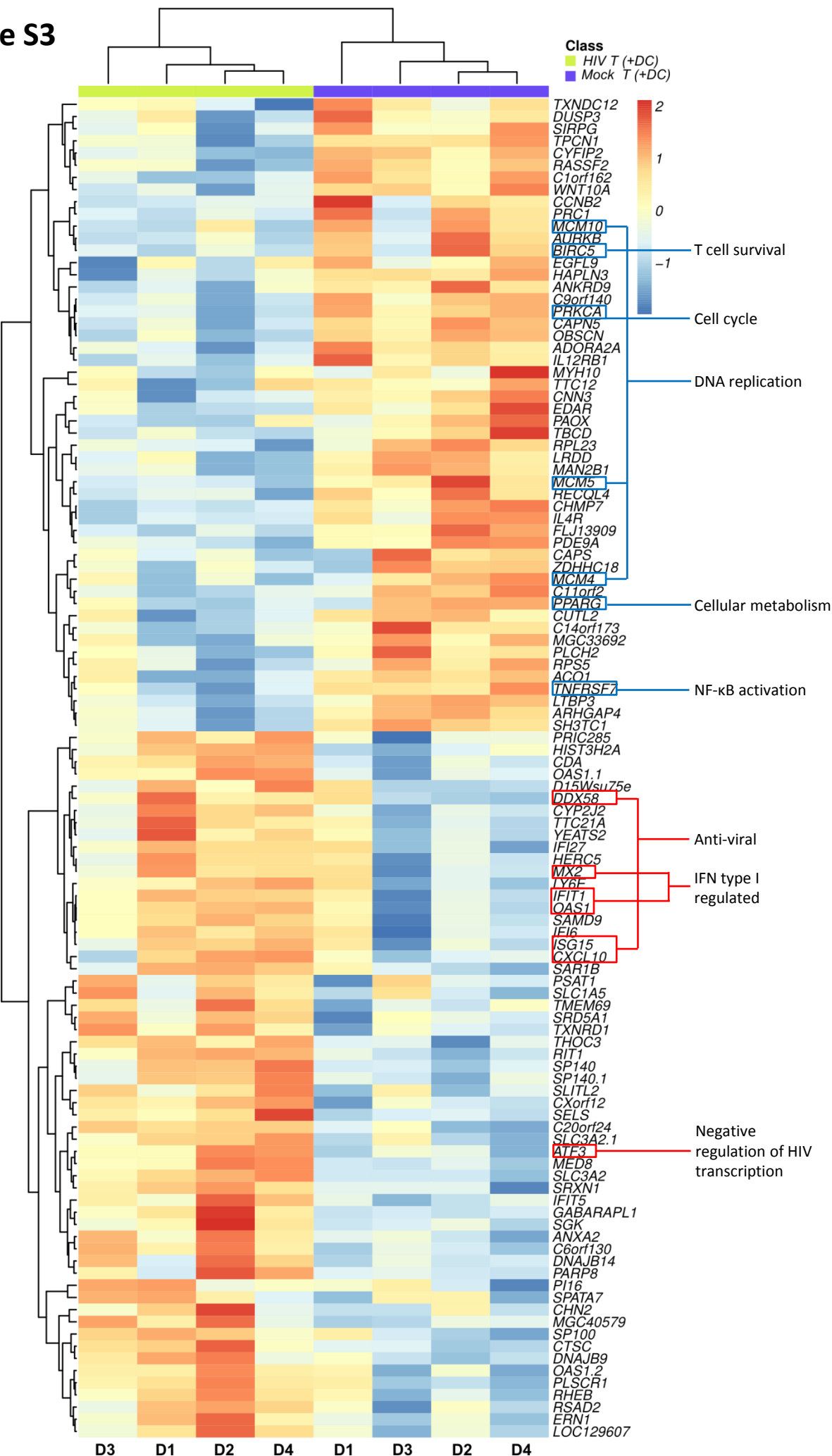

Supplement: Figure S3 — Top differentially expressed genes. Supervised clustering heatmap of the top differentially expressed genes resulting from comparing HIV T (+DC) and Mock T (+DC) samples after subtracting HIV T (CD4+ T cells cultured with HIV) and Mock T (CD4+ T cells cultured in media alone) from each group respectively. Genes were selected as differentially expressed based on Fold Change (≥1.5 fold up or down-regulation) and a p-value<0.05, following a moderate t test as implemented in the LIMMA package. The scale shows the level of gene expression where red and blue correspond to up and down-regulation respectively. (PDF) [file ppat.1003799.s003.pdf]
