# Supplementary material for: Myeloid Dendritic Cells Induce HIV-1 Latency in Non-proliferating CD4+ T Cells
Source: PLoS Pathog. 2013 Dec 5;9(12):e1003799. doi: 10.1371/journal.ppat.1003799 (PMC3855553; doi:10.1371/journal.ppat.1003799)
Supplement: Table S1 — Significant pathways. Significant pathways differentially expressed in HIV (+DC) relative to Mock T (+DC) after the subtraction of HIV T and Mock T respectively. Gene symbols are colour coded indicating either up-regulation (red) or down-regulation (blue). © 2000–2013 Ingenuity Systems, Inc. All rights reserved. (PDF) [file ppat.1003799.s004.pdf]

Table S1. Significant Pathways

Ingenuity Canonical Pathways	-log(p-value)	Ratio	Molecules
Mechanisms of Viral Exit from Host Cells	3.6E00	1.33E-01	VPS28,CHMP2A,ACTA2,LMNB2,LMNB1, PRKCA
Activation of IRF by Cytosolic Pattern Recognition Receptors	2.54E00	8.33E-02	IRF7,DDX58,ZBP1,TBK1,IFIT2,ISG15
Spermine and Spermidine Degradation I	2.53E00	1.43E-01	SAT1,PAOX
Role of RIG1-like Receptors in Antiviral Innate Immunity	2.52E00	1.02E-01	TRAF2,IRF7,DDX58,TBK1,TRIM25
Regulation of eIF4 and p70S6K Signaling	2.21E00	5.17E-02	RPS29,PABPC1,EIF2C4,RPS5,RPS9, EIF3J,PPP2R3B,EIF3L,EIF3K
Superpathway of Serine and Glycine Biosynthesis I	2.15E00	1.11E-01	PSAT1,SHMT1
Thioredoxin Pathway	2.15E00	2.5E-01	TXN,TXNRD1
Salvage Pathways of Pyrimidine Deoxyribonucleotides	2.15E00	1.11E-01	CDA,TK1
Endoplasmic Reticulum Stress Pathway	2.14E00	1.67E-01	TRAF2,ERN1,CASP7
Death Receptor Signaling	2.01E00	7.81E-02	TRAF2,TNFRSF25,TBK1,TNFRSF10A, CASP7
mTOR Signaling	1.95E00	4.76E-02	RPS29,RPS6KA6,RHEB,RPS5,RPS9, EIF3J,PPP2R3B,EIF3L,EIF3K,PRKCA
Folate Transformations I	1.78E00	6.25E-02	MTHFD2,SHMT1
NRF2-mediated Oxidative Stress Response	1.68E00	4.69E-02	ACTA2,HERPUD1,TXN,GCLM,DNAJB9, DNAJA1,DNAJB14,TXNRD1,PRKCA
Role of Pattern Recognition Receptors in Recognition of Bacteria and Viruses	1.67E00	5.66E-02	TLR1,OAS1,IRF7,OAS2,DDX58,PRKCA
Cell Cycle Control of Chromosomal Replication	1.65E00	9.68E-02	MCM5,MCM2,MCM4
Histamine Biosynthesis	1.65E00	3.33E-01	HDC
EIF2 Signaling	1.62E00	4.5E-02	RPS29,PABPC1,EIF2C4,RPS5,RPS9, EIF3J,RPL23,EIF3L,EIF3K
TWEAK Signaling	1.42E00	7.89E-02	TRAF2,TNFRSF25,CASP7
Interferon Signaling	1.39E00	8.33E-02	IFIT1,OAS1,IFI35
Threonine Degradation II	1.35E00	1.11E-01	GCAT
Putrescine Biosynthesis III	1.35E00	2.5E-01	ADC
Glycine Biosynthesis I	1.35E00	1.67E-01	SHMT1
Granzyme B Signaling	1.31E00	1.25E-01	LMNB2,LMNB1
Antigen Presentation Pathway	1.3E00	7.5E-02	HLA-E,CD74,TAPBP

Significant pathways differentially expressed in HIV T (+DC) relative to Mock T (+DC) after the subtraction of HIV T and Mock T respectively. Gene symbols are colour coded indicating either up-regulation (red) or down-regulation (blue).

© 2000-2013 Ingenuity Systems, Inc. All rights reserved.
