# Supplementary material for: Myeloid Dendritic Cells Induce HIV-1 Latency in Non-proliferating CD4+ T Cells
Source: PLoS Pathog. 2013 Dec 5;9(12):e1003799. doi: 10.1371/journal.ppat.1003799 (PMC3855553; doi:10.1371/journal.ppat.1003799)
Supplement: Table S2 — RT-PCR validated genes. Fold change obtained from either gene-array or RT-PCR representing the change in expression level for each gene in HIV T (+DC) relative to Mock T (+DC) after the subtraction of HIV T and Mock T respectively. (PDF) [file ppat.1003799.s005.pdf]

Table S2. RT-PCR validated genes

Symbol	Entrez Gene Name	Fold Change Arrays	RT-PCR
IFI27	interferon, alpha-inducible protein 27	6.35	3.06
IFIT1	interferon-induced protein with tetratricopeptide repeats 1	3.90	3.68
CXCL10	chemokine (C-X-C motif) ligand 10	2.89	4.70
OAS1	2',5'-oligoadenylate synthetase 1, 40/46kDa	2.87	2.53
RSAD2	radical S-adenosyl methionine domain containing 2	2.44	2.03
HERC5	hect domain and RLD 5	2.40	2.60
MX2	myxovirus (influenza virus) resistance 2 (mouse)	2.38	2.12
IFIT2	interferon-induced protein with tetratricopeptide repeats 2	2.16	2.22
IFI6	interferon, alpha-inducible protein 6	1.92	1.72
TXNRD1	Thioredoxin reductase 1, transcript variant 1	1.83	1.79
ATF3	activating transcription factor 3, transcript variant 4	1.76	1.46
DDIT3	DNA-damage-inducible transcript 3	1.70	1.41
IFI16	interferon, gamma-inducible protein 16	1.66	1.46
ERN1	endoplasmic reticulum to nucleus signalling 1, transcript variant 2	1.65	2.34
TRIM22	Tripartite motif-containing 22	1.61	1.22
IRF7	Interferon regulatory factor 7	1.58	1.60
H1F0	H1 histone family, member 0	1.52	1.79
GADD45A	growth arrest and DNA-damage-inducible, alpha	1.51	1.22
IL15	interleukin 15, transcript variant 1	1.43	1.24
KLF6	Kruppel-like factor 6, transcript variant 1	1.33	1.67
POLA1	polymerase (DNA directed), alpha 1	-1.07	-1.03
CDC2	cell division cycle 2, G1 to S and G2 to M, transcript variant 2	-1.29	-1.76
TNFRSF14	Tumor necrosis factor receptor superfamily, member 14	-1.28	1.06
ITGAL	Integrin, alpha L (antigen CD11A (p180))	-1.30	-1.17
WDR34	WD repeat domain 34	-1.31	-1.69
KIFC1	kinesin family member C1	-1.36	-1.82
E2F2	S phase, cell cycle progression, proliferation, G1/S phase transition, division	-1.41	-1.13
CDC20	CDC20 cell division cycle 20 homolog (S. cerevisiae)	-1.51	-3.23
EDAR	Ectodysplasin A receptor	-1.55	-1.57
UBE2C	ubiquitin-conjugating enzyme E2C	-1.55	-2.42
TYMS	thymidylate synthetase	-1.64	-2.23
TNFRSF25	Tumor necrosis factor recptor superfamily, member 25	-1.65	2.52
LRDD	Leucine-rich repeats and death domain containing	-1.70	-1.33
PRKCA	Protein kinase C, alpha	-1.74	-1.56
AURKB	aurora kinase B	-1.74	-2.14
CDCA5	cell division cycle associated 5	-1.83	-2.16
PDE9A	phosphodiesterase 9A	-1.85	1.52
IL4R	interleukin 4 receptor	-1.86	-1.58
BIRC5	baculoviral IAP repeat-containing 5 (survivin), transcript variant 1	-2.07	-2.92
TNFRSF7	CD27 molecule	-2.14	-1.41
MAN2B1	mannosidase, alpha, class 2B, member 1	-2.27	-1.25

Fold change obtained from either gene-array or RT-PCR represents the change in expression level for each gene in HIV T (+DC) relative to Mock T (+DC) after the subtraction of HIV T and Mock T respectively.
